# Clay-Coated Meshes with Superhydrophilicity and Underwater Superoleophobicity for Highly Efficient Oil/Water Separation

**DOI:** 10.3390/ma16124396

**Published:** 2023-06-15

**Authors:** Shaolin Yang, Cheng Zhen, Fangfang Li, Panpan Fu, Maohui Li, Youjun Lu, Zhilin Sheng

**Affiliations:** 1 School of Materials Science and Engineering, Ningxia Research Center of Silicon Target and Silicon-Carbon Negative Materials Engineering Technology, North Minzu University, Yinchuan 750021, China; slyang@nun.edu.cn (S.Y.); cheng_zhen_1998@163.com (C.Z.); fangfangli9@163.com (F.L.); 13669218402@163.com (P.F.); szl_djg@yeah.net (Z.S.); 2 School of Materials Science and Engineering, National and Local Joint Engineering Research Center of Advanced Carbon-Based Ceramics Preparation Technology, North Minzu University, Yinchuan 750021, China; youjunlu518@hotmail.com

**Keywords:** clay, oil/water separation, underwater superoleophobicity, stainless steel mesh, brush-coating

## Abstract

A novel clay-coated mesh was fabricated via a simple brush-coating method without the use of special equipment, chemical reagents, and complex chemical reactions and operation processes. Possessing superhydrophilicity and underwater superoleophobicity, the clay-coated mesh can be used for efficiently separating various light oil/water mixtures. The clay-coated mesh also exhibits excellent reusability, maintaining a high separation efficiency of 99.4% after 30 repeated separations of the kerosene/water mixture.

## 1. Introduction

Currently, an increasing amount of research is focusing on oil/water separation because of the increasing prevalence of oily wastewater and frequently occurring oil leakage accidents [1,2]. To date, various techniques, including in situ burning [3], air flotation [4], electrical/chemical coalescence [5], absorption [6], and membrane separation [7], have been developed for oily water disposal. Among them, membrane separation is the most favorable thanks to its advantages such as low energy consumption, easy operability, and high efficiency [8]. Membrane separation is realized using a superwetting membrane that can selectively remove one phase from the oil/water mixture.

Superhydrophobic-superoleophilic membranes have previously been utilized for oil/water separation [9,10]. However, superoleophilic membranes often suffer from fouling and even clogging caused by viscous oils during the oil permeation process. In addition, they are not suitable for separating light oil/water mixtures, which exist in most conditions. On the contrary, superhydrophilic and underwater superoleophobic (SUS) membranes can be used to effectively avoid oil fouling and realize light oil/water separation through discharging water under the driving force of gravity [11].

SUS membranes have been produced by coating hydrophilic materials on various porous substrates, including textiles [12], polymeric membranes [13], carbon cloths [14], and metal meshes [15]. Among them, stainless steel meshes (SSM) are the most widely used substrates owing to advantages such as their low cost, high mechanical strength and flexibility, and commercial availability [16]. To date, a variety of materials, such as nanostructured metal oxides/hydroxides [17], metal–organic frameworks [18], graphene oxides [19], polymers [20], and composites [21], have been coated on SSMs to produce SUS membranes. Nevertheless, the fabrication of these membranes is often hampered by the involvement of complex chemical reactions, toxic chemical reagents, special equipment, and complicated processes.

In this paper, we demonstrate a simple, cheap, and green method of fabricating a clay-coated mesh (CCM) through the brush coating of clay onto an SSM. Possessing superhydrophilicity and underwater superoleophobicity, the CCM can be used to separate various light oil/mixtures through the action of gravity, yielding a high separation efficiency of over 98.8%. The CCM also exhibits high recyclability, presenting a separation efficiency of 99.4% after 30 separation cycles.

## 2. Experimental Section

### 2.1. Materials

Clay and SSM were purchased from Taobao Market. Motor oil was supplied by FAW Mazda Motor Sales Co. Ltd., Changchun, China. Other organic liquids were purchased from Sinopharm Chemical Reagent Co., Ltd., Shanghai, China.

### 2.2. Preparation of CCMs

SSMs were first cropped into rectangles (30 mm × 30 mm) and cleaned with water. Clay was smashed and sifted by a sifter with a pore size of 0.1 mm. The sifted clay was mixed with water (m_clay_:m_water_ = 1:1) and stirred into a slurry. After the clay slurry was brushed onto the SSM, the air-dried CCM was repeatedly and thoroughly rinsed with water to desorb the excess clay. Finally, the derived CCM was dried for investigation.

### 2.3. Separation of Oil/Water Mixture

The separation setup was constructed by clamping the CCM between two glass tubes. Oil/water separation was performed by pouring the mixture of light oil and water into the upward-facing tubes and simultaneously collecting the filtrated water with a container placed below the lower tubes. Separation efficiency (*μ*) was computed using the following formula, *μ = m*_2_*/m*_1_ × 100%, where *m*_1_ is the mass of water in the mixture and *m*_2_ is that after separation.

### 2.4. Characterization

Scanning electron microscopy (SEM) was performed using a ZEISS SIGMA 500/VP field-emission scanning electron microscope (Oberkochen, Germany). X-ray diffraction (XRD) was conducted using a Rigaku RINTTTR III X-ray diffractometer (Tokyo, Japan). Contact angles (CAs) were measured using a POWEREACH JC200D2 optical contact angle measuring instrument (Shanghai, China).

## 3. Results and Discussion

**Figure 1:**
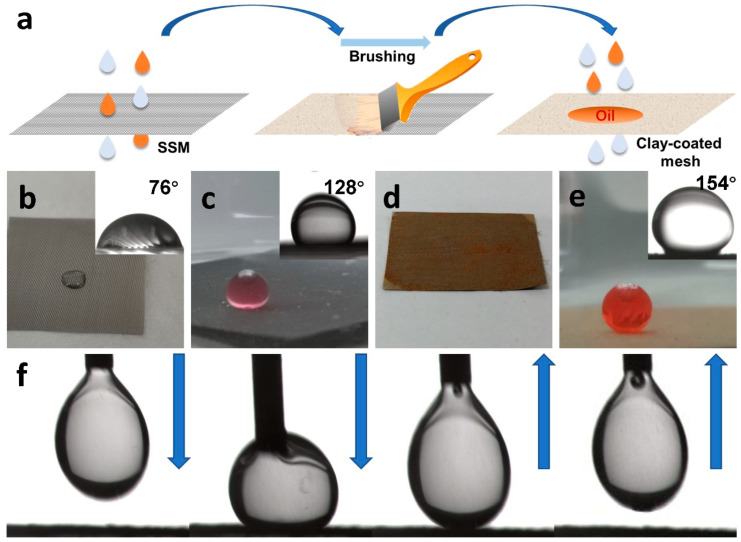
Fabrication process of the CCM (**a**); photographs of one drop of water in the air (**b**) and one drop of chlorobenzene in water on the bare SSM (inset: CA) (**c**); water dripping onto the CCM; (**d**) chlorobenzene droplet in water on the CCM (**e**); contact and detachment process of chlorobenzene droplet on the surface of the CCM (**f**).

The fabrication procedure of the CCM is illustrated in Figure 1a. No chemical agents, special devices, complex reactions, or tedious processes were involved. This method constitutes a mild, facile, inexpensive, and environmentally friendly approach to fabricating a superwetting membrane. As shown in Figure S1, the bare SSM is gray in coloration. After being coated with clay, the surface of the CCM becomes completely yellow, suggesting the uniform coating of the clay. The clay coating is physically attached to the surface of the SSM through strong van der Waals forces. The surface of the bare SSM presents a water CA of 76° (Figure 1b) and an underwater oil CA of 128° (Figure 1c). In contrast, the water droplets that drip onto the surface permeate into the CCM immediately (Figure 1d), while the chlorobenzene droplet maintain an almost spherical shape with a CA of 154° on the surface of the CCM immersed in water (Figure 1e), reflecting the SUS nature of the CCM. As shown in Figure S2, the chlorobenzene droplet on the surface of the CCM still preserved its original, nearly spherical shape without a noticeable change after being immersed in water for several days, suggesting the excellent underwater wettability stability of the CCM. The oil can easily be removed from the surface of the CCM and even compressed onto the surface underwater (Figure 1f), indicating the low oil adhesion of the CCM at the oil–water–solid interface. 

**Figure 2:**
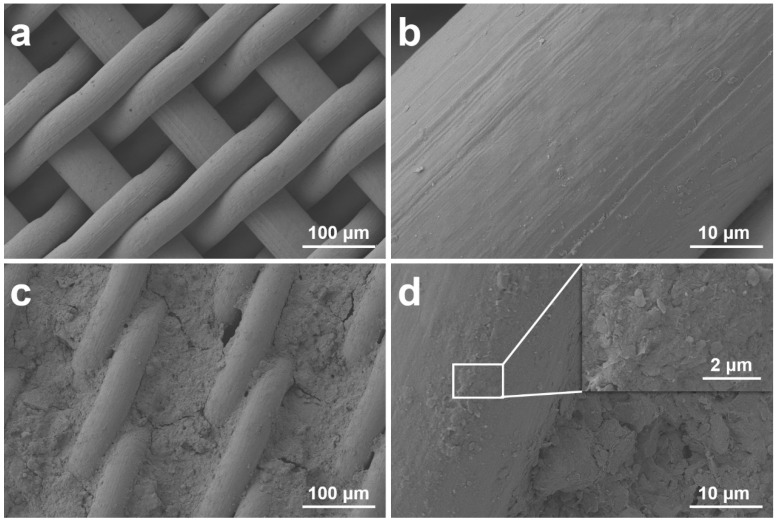
SEM images of SSM (**a**,**b**) and CCM (**c**,**d**) at different levels of magnification.

Figure 2 shows SEM images of the bare SSM and CCM. The SSM consists of intercrossed steel wires (Figure 2a) with a smooth surface (Figure 2b). As for the CCM, most of the coated clay fills in the grooves between the neighboring wires of the mesh (Figure 2c). Many fissures and pores with a size of 1~3 μm (Figure S3) exist on the coated clay, which is beneficial to the penetration of water through the mesh. The higher-magnification images shown in Figure 2d reveal that the wire surface is covered with a thin layer of clay. The coated clay formed a coarse microstructure, which is beneficial to the SUS property of the resultant mesh.

**Figure 3:**
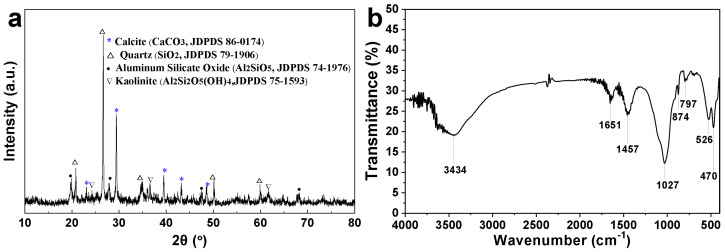
XRD pattern (**a**) and FTIR spectrum of the CCM (**b**).

As shown in the XRD pattern of the CCM displayed in Figure 3a, the main compositions of the coated clay are calcite, quartz, aluminum silicate oxide, and kaolinite. Figure 3b displays the FTIR spectrum of the CCM. The absorption peaks at 1027 and 470 cm^−1^ are associated with the Si-O vibration of quartz, aluminum silicate oxide, and kaolinite [22]. The peaks at 797 and 526 cm^−1^ correspond to the Al-O bonds of aluminum silicate oxide and kaolinite [23]. The peaks at 1457 cm^−1^ correspond to the CO_3_^2−^ of calcite [24]. The peaks at 3434, 1651, and 874 cm^−1^ originate from the O-H bending of kaolinite [22]. The abundance of hydroxyl groups and the microstructures of the coated clay grant the CCM superhydrophilic properties. Such superhydrophilic microstructures can trap water, which is essential for the underwater oil repellency of the CCM. 

Considering its SUS wettability, the CCM can be applied to light oil/water separation. Figure 3a displays the thorough permeation of water through the superhydrophilic CCM and the rejection of oil (dyed red) on the top of the underwater superoleophobic mesh after the mixture of the light oil and water was decanted into the upper tube of the separation apparatus. The separation process was merely completed under the driving force of gravity without the influence of another external force. No visible red oil was mixed in the harvest water, indicating that the CCM can be utilized for light oil/water separation. In comparison, neither oil nor water were blocked by the bare SSM (Figure S4). The separability of the CCM was evaluated via separating various light oil/water mixtures. As displayed in Figure 3b, the separation efficiencies for all mixtures can are higher than 98.8%, even reaching 99.7% for the separation of the cyclohexane/water mixture, reflecting the excellent separation ability of the CCM. These separation efficiencies are higher than most previously reported SUS meshes such as graphene-oxide-coated meshes (≥98.2%) [15], polydopamine/attapulgite-nanorod-deposited meshes (≥97.5%) [25], NaA zeolite/copper meshes (98.6%) [26], and cement–alumina-coated meshes (≥98.3%) [27]. The reusability of the CCM was determined by repeating the kerosene/water separation 30 times, for which the CCM was flushed with water after each separation cycle. The CCM could still maintain a high separation efficiency of 99.4% after 30 reuse cycles (Figure 3c), indicating its superior recyclability with respect to oil/water separation. In addition, the CCM still preserved its original morphology (Figure S5a) and underwater superoleophobicity (with an oil CA of 153° (Figure S5b)), reflecting the excellent stability of the coating.

**Figure 4:**
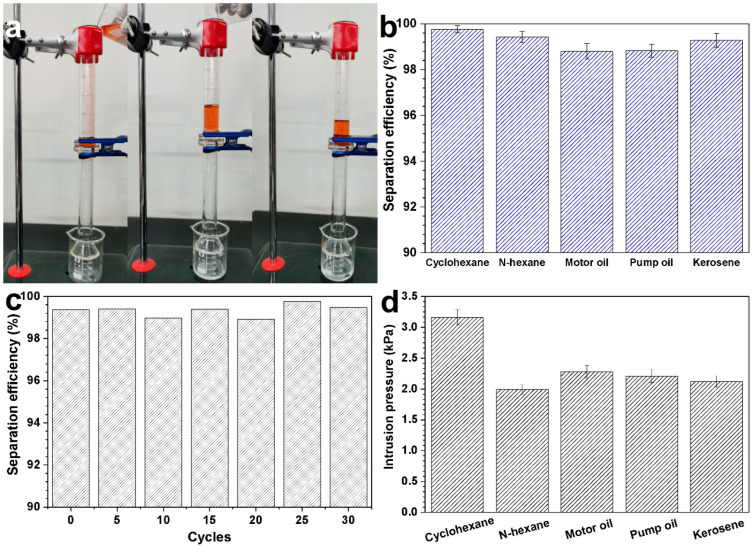
Oil/water separation process with the CCM (**a**); separation efficiencies of the CCM for the mixtures of various oils (**b**); variation of the separation efficiency for kerosene/water separation with an increasing number of cycles (**c**); intrusion pressures of the water-prewetted CCM for various types of oil (**d**).

Oil intrusion pressure, i.e., the highest oil pressure that a mesh can support, is also an important indicator of the capacity for oil/water separation. Therefore, the intrusion pressure of the water-prewetted CCM for various types of oil was measured, and the value was calculated using the following equation: *p = ρgh*_max_. Here, *p* is the intrusion pressure, *ρ* is the oil density, *g* is the acceleration of gravity, and *h*_max_ is the maximal height of oil that cannot permeate the mesh. The maximum-bearable heights for kerosene, cyclohexane, n-hexane, motor oil, and pump oil are 40.3, 26.1, 35.3, 24.8, and 25.2 cm, respectively, corresponding to the intrusion pressures of 3.16, 1.99, 2.28, 2.21, and 2.12 kPa (Figure 4d). The average intrusion pressure of the CCM for these oils is 2.35 kPa, reflecting the outstanding oil-bearing capacity of the CCM.

## 4. Conclusions

In summary, a novel SUS CCM was fabricated via a simple brush-coating method. The SUS wettability achieved can be ascribed to the coated clay’s superhydrophilicity and micro-rough coating surface. The CCM exhibits excellent capacity for the separation of light oil/water mixtures with high efficiency and recyclability. The CCM also has a high average oil intrusion pressure of 2.35 kPa. Its advantages in terms of fabrication and its superior behavior exhibit the CCM’s potential practical applications in oily water treatment.

## Data Availability

The data presented in this study are available on request from the corresponding author.

## Supplementary Materials

The following supporting information can be downloaded at: https://www.mdpi.com/article/10.3390/ma16124396/s1.
