# Supplementary material for: Clay-Coated Meshes with Superhydrophilicity and Underwater Superoleophobicity for Highly Efficient Oil/Water Separation
Source: Materials (Basel). 2023 Jun 15;16(12):4396. doi: 10.3390/ma16124396 (PMC10304548; doi:10.3390/ma16124396)
Supplement: Supplementary file 1 [file materials-16-04396-s001.zip › materials-2426592-supplementary.pdf]

Supplementary Information for

Clay-Coated Meshes with Superhydrophilicity and Underwater Superoleophobicity for Highly Efficient Oil/Water Separation

Shaolin Yang [1], Cheng Zhen [1], Fangfang Li [1], Panpan Fu [1], Maohui Li [2,*], Youjun Lu [2], Zhilin Sheng [1]

[1] School of Materials Science and Engineering, Ningxia Research Center of Silicon Target and Silicon-Carbon Negative Materials Engineering Technology, North Minzu University, Yinchuan 750021, China

[2] School of Materials Science and Engineering, National and Local Joint Engineering Research Center of Advanced Carbon-Based Ceramics Preparation Technology, North Minzu University, Yinchuan 750021, China

* Correspondence: lmnlmb@nmu.edu.cn

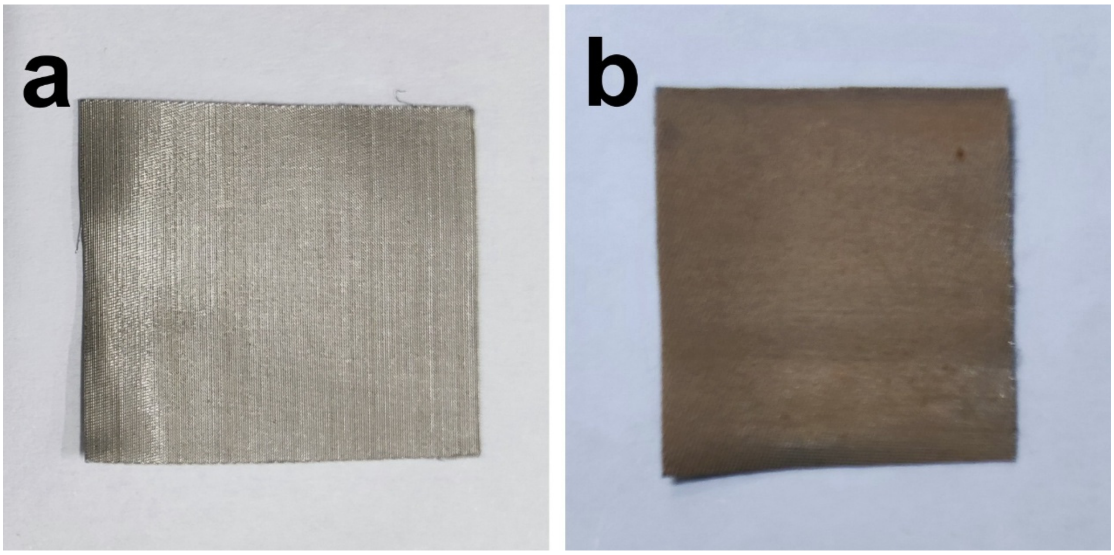

Figure S1. photographs of bare SSM (a) and CCM (b).

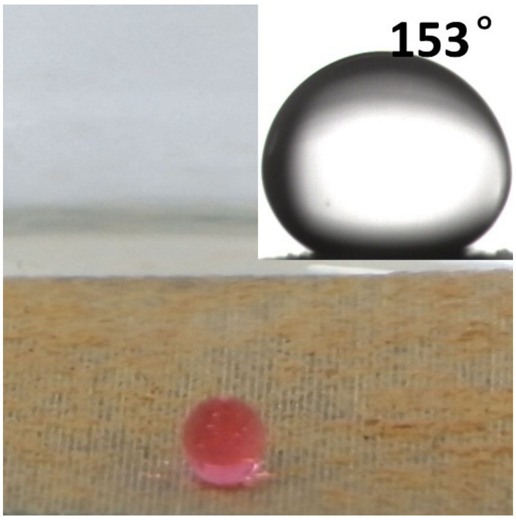

Figure S2. chlorobenzene droplets on the CCM under water for several days.

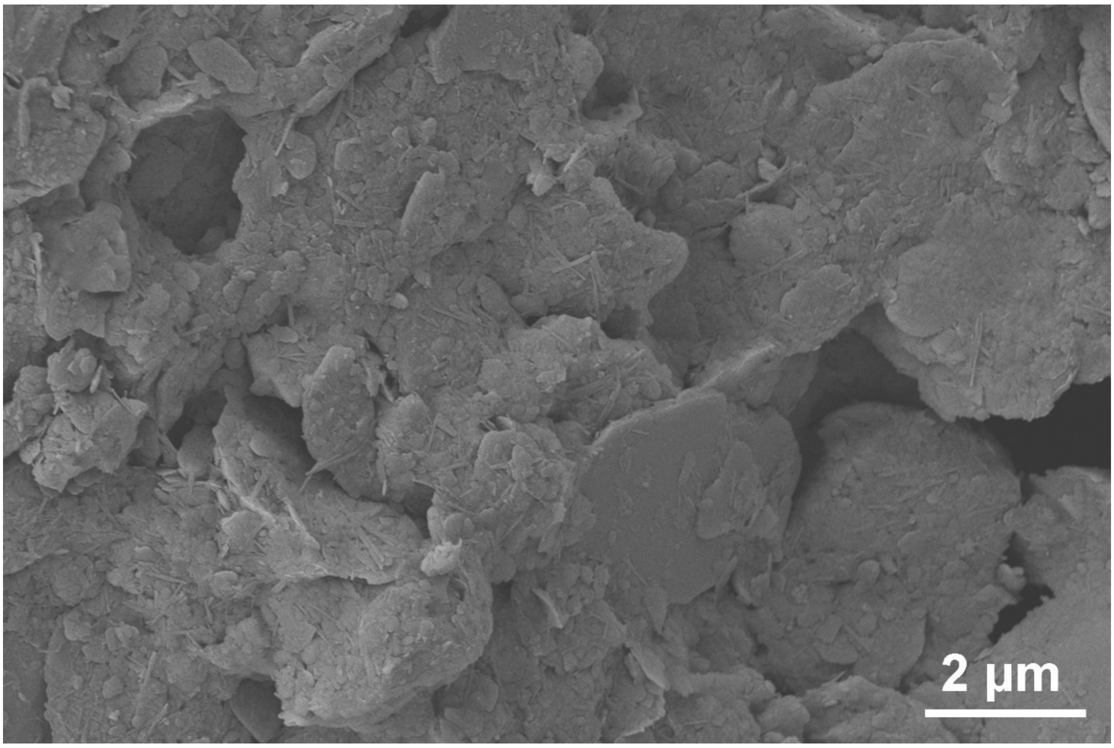

Figure S3. High magnification SEM image of the CCM.

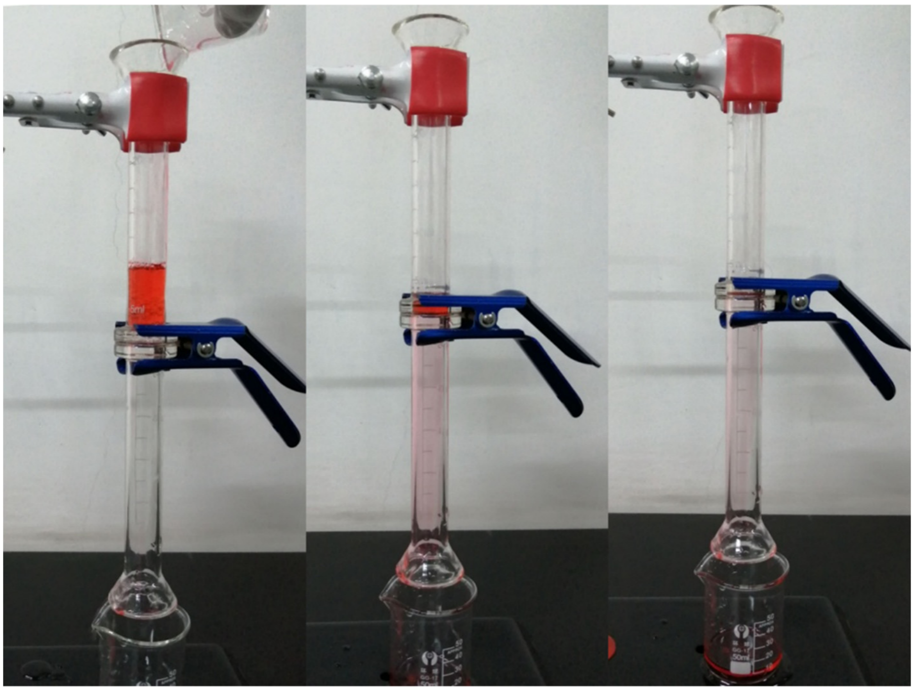

Figure S4. Separation process oil/water mixture with the bare SSM.

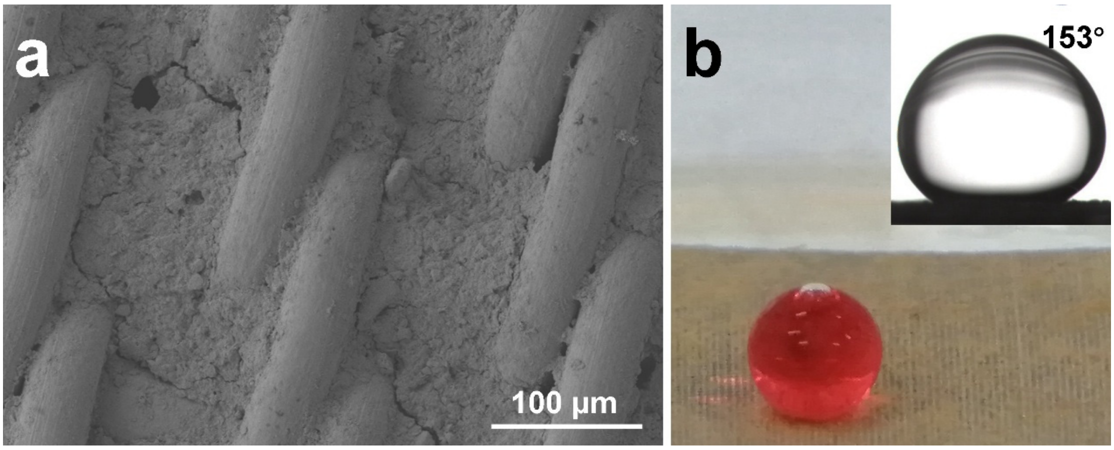

Figure S5. SEM (a) and underwater superoleophobicity of the CCM after it was repeated used for 30 cycles (b).
